# Supplementary material for: Comparative Pan-Genome Analysis of Oral Veillonella Species
Source: Microorganisms. 2021 Aug 20;9(8):1775. doi: 10.3390/microorganisms9081775 (PMC8400620; doi:10.3390/microorganisms9081775)
Supplement: Supplementary file 1 [file microorganisms-09-01775-s001.zip › Suppl-Table 1.pdf]

MAJOR_KEGG_CATEGORY	KEGG SUB-CATEGORY	KEGG PATHWAY	CORE	ACCESSORY	UNIQUE
Cellular_Processes	Cell_growth_and_death	04112 Cell cycle - Caulobacter [PATH:ko04112]	9	0	0
	Cell_motility	02030 Bacterial chemotaxis [PATH:ko02030]	1	0	0
	Cellular_commiunity	04510 Focal adhesion [PATH:ko04510]	0	0	3
		04146 Peroxisome [PATH:ko04146]	1	3	0
Environmental_Information_Processing	Membrane_transport	02010 ABC transporters [PATH:ko02010]	58	22	6
		02060 Phosphotransferase system (PTS) [PATH:ko02060]	5	0	0
		03070 Bacterial secretion system [PATH:ko03070]	12	2	1
	Signal_transduction	02020 Two-component system [PATH:ko02020]	17	6	2
		04011 MAPK signaling pathway - yeast [PATH:ko04011]	1	0	0
		04066 HIF-1 signaling pathway [PATH:ko04066]	2	0	0
		04068 FoxO signaling pathway [PATH:ko04068]	0	2	0
		04070 Phosphatidylinositol signaling system [PATH:ko04070]	1	0	0
		04151 PI3K-Akt signaling pathway [PATH:ko04151]	0	0	3
		04152 AMPK signaling pathway [PATH:ko04152]	0	1	0
	Signaling_molecules_and_interaction	04512 ECM-receptor interaction [PATH:ko04512]	0	1	3
Genetic_Information_Processing	Folding_sorting_and_degradation	03018 RNA degradation [PATH:ko03018]	12	1	0
		03060 Protein export [PATH:ko03060]	13	0	0
		04122 Sulfur relay system [PATH:ko04122]	4	0	2
	Replication_and_repair	03030 DNA replication [PATH:ko03030]	13	0	0
		03410 Base excision repair [PATH:ko03410]	8	0	0
		03420 Nucleotide excision repair [PATH:ko03420]	8	0	1
		03430 Mismatch repair [PATH:ko03430]	16	0	1
		03440 Homologous recombination [PATH:ko03440]	18	1	1
	Transcription	03020 RNA polymerase [PATH:ko03020]	4	0	0
	Translation	00970 Aminoacyl-tRNA biosynthesis [PATH:ko00970]	26	0	0
		03008 Ribosome biogenesis in eukaryotes [PATH:ko03008]	1	0	0
		03010 Ribosome [PATH:ko03010]	51	1	0
Human_Diseases	Cancers	05200 Pathways in cancer [PATH:ko05200]	1	0	1
		05203 Viral carcinogenesis [PATH:ko05203]	1	0	0
		05205 Proteoglycans in cancer [PATH:ko05205]	1	0	0
		05206 MicroRNAs in cancer [PATH:ko05206]	1	1	6
		05211 Renal cell carcinoma [PATH:ko05211]	1	0	0
		05230 Central carbon metabolism in cancer [PATH:ko05230]	1	1	0
	Drug_resistance	01501 beta-Lactam resistance [PATH:ko01501]	10	3	0
		01502 Vancomycin resistance [PATH:ko01502]	5	0	0
		01503 Cationic antimicrobial peptide (CAMP) resistance [PATH:ko01503]	6	1	0

	Endocrine_and_metabolic_diseases	04930 Type II diabetes mellitus [PATH:ko04930]	1	0	0
		04940 Type I diabetes mellitus [PATH:ko04940]	1	0	0
		05111 Vibrio cholerae pathogenic cycle [PATH:ko05111]	0	1	0
		05120 Epithelial cell signaling in Helicobacter pylori infection [PATH:ko05120]	2	0	0
		05132 Salmonella infection [PATH:ko05132]	1	0	0
		05133 Pertussis [PATH:ko05133]	0	1	2
		05134 Legionellosis [PATH:ko05134]	2	0	0
		05146 Amoebiasis [PATH:ko05146]	0	0	3
		05152 Tuberculosis [PATH:ko05152]	2	1	0
	Neurodegenerative_diseases	05010 Alzheimer's disease [PATH:ko05010]	1	0	0
		05014 Amyotrophic lateral sclerosis (ALS) [PATH:ko05014]	0	1	0
		05016 Huntington's disease [PATH:ko05016]	0	1	0
Metabolism	Amino_acid_metabolism	00250 Alanine, aspartate and glutamate metabolism [PATH:ko00250]	14	2	0
		00260 Glycine, serine and threonine metabolism [PATH:ko00260]	16	0	0
		00270 Cysteine and methionine metabolism [PATH:ko00270]	25	1	6
		00280 Valine, leucine and isoleucine degradation [PATH:ko00280]	7	1	0
		00290 Valine, leucine and isoleucine biosynthesis [PATH:ko00290]	10	2	0
		00300 Lysine biosynthesis [PATH:ko00300]	12	0	0
		00310 Lysine degradation [PATH:ko00310]	2	1	0
		00330 Arginine and proline metabolism [PATH:ko00330]	12	6	0
		00340 Histidine metabolism [PATH:ko00340]	1	9	0
		00350 Tyrosine metabolism [PATH:ko00350]	2	2	1
		00360 Phenylalanine metabolism [PATH:ko00360]	4	1	0
		00380 Tryptophan metabolism [PATH:ko00380]	0	2	0
		00400 Phenylalanine, tyrosine and tryptophan biosynthesis [PATH:ko00400]	18	2	0
	Biosynthesis_of_other_secondary_metabolites	00261 Monobactam biosynthesis [PATH:ko00261]	4	0	0
		00311 Penicillin and cephalosporin biosynthesis [PATH:ko00311]	1	0	0
		00332 Carbapenem biosynthesis [PATH:ko00332]	2	0	0
		00401 Novobiocin biosynthesis [PATH:ko00401]	1	2	0
		00521 Streptomycin biosynthesis [PATH:ko00521]	1	0	4
		00950 Isoquinoline alkaloid biosynthesis [PATH:ko00950]	2	0	0
		00960 Tropane, piperidine and pyridine alkaloid biosynthesis [PATH:ko00960]	3	1	0
	Carbohydrate_metabolism	00010 Glycolysis / Gluconeogenesis [PATH:ko00010]	13	3	1
		00020 Citrate cycle (TCA cycle) [PATH:ko00020]	10	0	1
		00030 Pentose phosphate pathway [PATH:ko00030]	15	2	0
		00040 Pentose and glucuronate interconversions [PATH:ko00040]	3	0	0
		00051 Fructose and mannose metabolism [PATH:ko00051]	6	1	0

		00052 Galactose metabolism [PATH:ko00052]	4	2	0
		00053 Ascorbate and aldarate metabolism [PATH:ko00053]	4	0	0
		00500 Starch and sucrose metabolism [PATH:ko00500]	3	1	0
		00520 Amino sugar and nucleotide sugar metabolism [PATH:ko00520]	11	5	0
		00562 Inositol phosphate metabolism [PATH:ko00562]	1	0	0
		00620 Pyruvate metabolism [PATH:ko00620]	20	4	4
		00630 Glyoxylate and dicarboxylate metabolism [PATH:ko00630]	14	3	1
		00640 Propanoate metabolism [PATH:ko00640]	13	1	1
		00650 Butanoate metabolism [PATH:ko00650]	6	4	2
		00660 C5-Branched dibasic acid metabolism [PATH:ko00660]	5	2	0
	Energy_metabolism	00190 Oxidative phosphorylation [PATH:ko00190]	15	0	0
		00195 Photosynthesis [PATH:ko00195]	8	0	0
		00680 Methane metabolism [PATH:ko00680]	12	1	0
		00710 Carbon fixation in photosynthetic organisms [PATH:ko00710]	9	0	0
		00720 Carbon fixation pathways in prokaryotes [PATH:ko00720]	23	1	1
		00910 Nitrogen metabolism [PATH:ko00910]	12	5	0
		00920 Sulfur metabolism [PATH:ko00920]	6	0	0
	Glycan_biosynthesis_and_metabolism	00510 N-Glycan biosynthesis [PATH:ko00510]	1	0	0
		00531 Glycosaminoglycan degradation [PATH:ko00531]	0	3	0
		00540 Lipopolysaccharide biosynthesis [PATH:ko00540]	17	0	1
		00550 Peptidoglycan biosynthesis [PATH:ko00550]	12	0	3
	Lipid_metabolism	00061 Fatty acid biosynthesis [PATH:ko00061]	7	1	0
		00071 Fatty acid degradation [PATH:ko00071]	0	3	1
		00072 Synthesis and degradation of ketone bodies [PATH:ko00072]	0	1	0
		00561 Glycerolipid metabolism [PATH:ko00561]	6	0	0
		00564 Glycerophospholipid metabolism [PATH:ko00564]	8	0	1
		00565 Ether lipid metabolism [PATH:ko00565]	0	0	0
		00590 Arachidonic acid metabolism [PATH:ko00590]	0	1	0
		01040 Biosynthesis of unsaturated fatty acids [PATH:ko01040]	1	0	0
	Metabolism_of_cofactors_and_vitamins	00130 Ubiquinone and other terpenoid-quinone biosynthesis [PATH:ko00130]	8	1	0
		00670 One carbon pool by folate [PATH:ko00670]	10	0	0
		00730 Thiamine metabolism [PATH:ko00730]	4	3	4
		00740 Riboflavin metabolism [PATH:ko00740]	5	0	0
		00750 Vitamin B6 metabolism [PATH:ko00750]	2	0	0
		00760 Nicotinate and nicotinamide metabolism [PATH:ko00760]	6	0	0
		00770 Pantothenate and CoA biosynthesis [PATH:ko00770]	10	5	0
		00780 Biotin metabolism [PATH:ko00780]	10	0	0

		00790 Folate biosynthesis [PATH:ko00790]	10	3	0
		00860 Porphyrin and chlorophyll metabolism [PATH:ko00860]	36	6	0
	Metabolism_of_other_amino_acids	00410 beta-Alanine metabolism [PATH:ko00410]	1	3	0
		00430 Taurine and hypotaurine metabolism [PATH:ko00430]	3	0	0
		00440 Phosphonate and phosphinate metabolism [PATH:ko00440]	1	1	0
		00450 Selenocompound metabolism [PATH:ko00450]	8	1	0
		00460 Cyanoamino acid metabolism [PATH:ko00460]	2	0	0
		00471 D-Glutamine and D-glutamate metabolism [PATH:ko00471]	3	0	0
		00472 D-Arginine and D-ornithine metabolism [PATH:ko00472]	1	0	0
		00473 D-Alanine metabolism [PATH:ko00473]	3	0	0
		00480 Glutathione metabolism [PATH:ko00480]	2	2	0
	Metabolism_of_terpenoids_and_polyketides	00523 Polyketide sugar unit biosynthesis [PATH:ko00523]	0	0	4
		00900 Terpenoid backbone biosynthesis [PATH:ko00900]	9	4	0
		00908 Zeatin biosynthesis [PATH:ko00908]	1	0	0
		01051 Biosynthesis of ansamycins [PATH:ko01051]	2	0	0
		01053 Biosynthesis of siderophore group nonribosomal peptides [PATH:ko01053]	1	0	0
		01055 Biosynthesis of vancomycin group antibiotics [PATH:ko01055]	0	0	1
	Nucleotide_metabolism	00230 Purine metabolism [PATH:ko00230]	45	5	0
		00240 Pyrimidine metabolism [PATH:ko00240]	34	3	1
	Overview	01200 Carbon metabolism [PATH:ko01200]	48	5	1
		01210 2-Oxocarboxylic acid metabolism [PATH:ko01210]	14	5	1
		01212 Fatty acid metabolism [PATH:ko01212]	7	2	0
		01220 Degradation of aromatic compounds [PATH:ko01220]	2	2	1
		01230 Biosynthesis of amino acids [PATH:ko01230]	75	17	1
	Xenobiotics_biodegradation_and_metabolism	00362 Benzoate degradation [PATH:ko00362]	2	2	0
		00621 Dioxin degradation [PATH:ko00621]	1	0	0
		00622 Xylene degradation [PATH:ko00622]	1	0	0
		00625 Chloroalkane and chloroalkene degradation [PATH:ko00625]	0	1	1
		00626 Naphthalene degradation [PATH:ko00626]	0	1	1
		00627 Aminobenzoate degradation [PATH:ko00627]	0	1	0
		00633 Nitrotoluene degradation [PATH:ko00633]	4	0	0
		00983 Drug metabolism - other enzymes [PATH:ko00983]	4	1	0
Organismal_Systems	Digestive_system	04974 Protein digestion and absorption [PATH:ko04974]	0	0	3
		04976 Bile secretion [PATH:ko04976]	1	0	0
	Endocrine_system	03320 PPAR signaling pathway [PATH:ko03320]	0	1	0
		04918 Thyroid hormone synthesis [PATH:ko04918]	0	1	0
		04920 Adipocytokine signaling pathway [PATH:ko04920]	0	1	0

		04922 Glucagon signaling pathway [PATH:ko04922]	2	0	0
	Immune_system	04611 Platelet activation [PATH:ko04611]	0	0	2
	Nervous_system	04724 Glutamatergic synapse [PATH:ko04724]	1	0	0
		04727 GABAergic synapse [PATH:ko04727]	1	0	0
