# Supplementary material for: Comparative Pan-Genome Analysis of Oral Veillonella Species
Source: Microorganisms. 2021 Aug 20;9(8):1775. doi: 10.3390/microorganisms9081775 (PMC8400620; doi:10.3390/microorganisms9081775)
Supplement: Supplementary file 1 [file microorganisms-09-01775-s001.zip › Suppl-Table 2.pdf]

No.	gene	type	cluster	genome	genomic_accession	start	end
1	PQL18695.1	unique	2777	1	PPDE01000001.1	33371	33601
2	PQL18659.1	unique	2865	1	PPDE01000002.1	46853	47041
3	PQL18387.1	unique	1113	1	PPDE01000003.1	26816	27775
4	PQL18389.1	unique	1365	1	PPDE01000003.1	28247	29080
5	PQL18630.1	unique	2193	1	PPDE01000003.1	292731	293189
6	PQL18640.1	unique	2428	1	PPDE01000003.1	292244	292624
7	PQL18335.1	unique	1953	1	PPDE01000004.1	1477	2046
8	PQL18360.1	unique	862	1	PPDE01000004.1	25369	26484
9	PQL18237.1	unique	1284	1	PPDE01000005.1	49118	49993
10	PQL18238.1	unique	455	1	PPDE01000005.1	50187	51620
11	PQL18328.1	unique	2853	1	PPDE01000005.1	143875	144069
12	PQL18116.1	unique	1800	1	PPDE01000006.1	5837	6463
13	PQL18117.1	unique	1922	1	PPDE01000006.1	6378	6953
14	PQL18119.1	unique	1992	1	PPDE01000006.1	7339	7890
15	PQL18120.1	unique	2565	1	PPDE01000006.1	9187	9510
16	PQL18121.1	unique	1539	1	PPDE01000006.1	9539	10294
17	PQL18122.1	unique	2384	1	PPDE01000006.1	10296	10691
18	PQL18123.1	unique	35	1	PPDE01000006.1	10688	14758
19	PQL18124.1	unique	2559	1	PPDE01000006.1	14762	15088
20	PQL18125.1	unique	1847	1	PPDE01000006.1	15072	15677
21	PQL18126.1	unique	2539	1	PPDE01000006.1	15679	16011
22	PQL18127.1	unique	60	1	PPDE01000006.1	16014	19226
23	PQL18128.1	unique	2595	1	PPDE01000006.1	19216	19527
24	PQL18129.1	unique	2663	1	PPDE01000006.1	19545	19826
25	PQL18130.1	unique	2168	1	PPDE01000006.1	20805	21275
26	PQL18131.1	unique	1689	1	PPDE01000006.1	21278	21955
27	PQL18132.1	unique	2620	1	PPDE01000006.1	21952	22251
28	PQL18133.1	unique	2541	1	PPDE01000006.1	22316	22648
29	PQL18134.1	unique	230	1	PPDE01000006.1	22666	24567
30	PQL18135.1	unique	403	1	PPDE01000006.1	24570	26072
31	PQL18136.1	unique	2811	1	PPDE01000006.1	26057	26269
32	PQL18137.1	unique	217	1	PPDE01000006.1	26281	28218
33	PQL18138.1	unique	2067	1	PPDE01000006.1	28148	28657
34	PQL18139.1	unique	2373	1	PPDE01000006.1	29037	29438
35	PQL18140.1	unique	2614	1	PPDE01000006.1	29469	29771
36	PQL18142.1	unique	2625	1	PPDE01000006.1	32048	32347
37	PQL18143.1	unique	102	1	PPDE01000006.1	32663	35128
38	PQL18144.1	unique	211	1	PPDE01000006.1	35145	37112
39	PQL18145.1	unique	1795	1	PPDE01000006.1	37125	37751

40	PQL18146.1	unique	791	1	PPDE01000006.1	37754	38917
41	PQL18147.1	unique	2261	1	PPDE01000006.1	38917	39354
42	PQL18148.1	unique	2698	1	PPDE01000006.1	39380	39643
43	PQL18149.1	unique	2731	1	PPDE01000006.1	39649	39900
44	PQL18151.1	unique	1852	1	PPDE01000006.1	41356	41958
45	PQL18152.1	unique	2154	1	PPDE01000006.1	42046	42519
46	PQL18153.1	unique	2870	1	PPDE01000006.1	42516	42701
47	PQL18154.1	unique	2778	1	PPDE01000006.1	42685	42915
48	PQL18157.1	unique	2372	1	PPDE01000006.1	44199	44600
49	PQL18194.1	unique	2795	1	PPDE01000006.1	29775	29996
50	PQL17450.1	unique	2845	1	PPDE01000008.1	84653	84850
51	PQL17528.1	unique	311	1	PPDE01000008.1	172780	174471
52	PQL17529.1	unique	147	1	PPDE01000008.1	174473	176680
53	PQL17530.1	unique	611	1	PPDE01000008.1	176682	177968
54	PQL17531.1	unique	96	1	PPDE01000008.1	177961	180477
55	PQL17532.1	unique	1673	1	PPDE01000008.1	180527	181213
56	PQL20889.1	unique	1192	2	PPDB01000001.1	45245	46174
57	PQL20890.1	unique	1058	2	PPDB01000001.1	46266	47258
58	PQL20918.1	unique	2075	2	PPDB01000001.1	75051	75557
59	PQL20929.1	unique	890	2	PPDB01000001.1	87883	88974
60	PQL20970.1	unique	489	2	PPDB01000001.1	139835	141223
61	PQL20972.1	unique	260	2	PPDB01000001.1	142347	144170
62	PQL20973.1	unique	2166	2	PPDB01000001.1	144170	144640
63	PQL20974.1	unique	446	2	PPDB01000001.1	145053	146492
64	PQL21017.1	unique	2824	2	PPDB01000001.1	204408	204614
65	PQL21018.1	unique	1484	2	PPDB01000001.1	204904	205680
66	PQL21019.1	unique	2876	2	PPDB01000001.1	205837	206019
67	PQL21021.1	unique	2786	2	PPDB01000001.1	206251	206475
68	PQL21030.1	unique	2199	2	PPDB01000001.1	211080	211538
69	PQL21034.1	unique	1861	2	PPDB01000001.1	213070	213669
70	PQL21035.1	unique	277	2	PPDB01000001.1	213666	215414
71	PQL21058.1	unique	1951	2	PPDB01000001.1	235344	235913
72	PQL21059.1	unique	2229	2	PPDB01000001.1	235903	236352
73	PQL21236.1	unique	939	2	PPDB01000001.1	146544	147596
74	PQL20246.1	unique	2602	2	PPDB01000003.1	353305	353613
75	PQL20306.1	unique	1174	2	PPDB01000003.1	421300	422235
76	PQL20308.1	unique	1097	2	PPDB01000003.1	423888	424859
77	PQL20346.1	unique	528	2	PPDB01000003.1	468897	470249
78	PQL20349.1	unique	1767	2	PPDB01000003.1	474711	475349
79	PQL20379.1	unique	2131	2	PPDB01000003.1	507260	507745

80	PQL20416.1	unique	2299	2	PPDB01000003.1	557344	557769
81	PQL20417.1	unique	918	2	PPDB01000003.1	557879	558949
82	PQL20448.1	unique	563	2	PPDB01000003.1	555534	556862
83	WP_005385388.1	unique	1366	3	NZ_GG667604.1	453962	454792
84	WP_005385391.1	unique	2830	3	NZ_GG667604.1	456295	456498
85	WP_005385394.1	unique	2769	3	NZ_GG667604.1	456843	457076
86	WP_005385396.1	unique	2799	3	NZ_GG667604.1	457128	457346
87	WP_005385397.1	unique	2887	3	NZ_GG667604.1	457339	457518
88	WP_005385398.1	unique	2916	3	NZ_GG667604.1	457536	457685
89	WP_005385399.1	unique	2524	3	NZ_GG667604.1	457660	458001
90	WP_005385633.1	unique	2079	3	NZ_GG667604.1	607192	607695
91	WP_005385878.1	unique	2852	3	NZ_GG667604.1	741688	741882
92	WP_005386155.1	unique	701	3	NZ_GG667604.1	916330	917553
93	WP_005386157.1	unique	292	3	NZ_GG667604.1	920920	922650
94	WP_005386158.1	unique	272	3	NZ_GG667604.1	922647	924404
95	WP_005386167.1	unique	543	3	NZ_GG667604.1	932709	934052
96	WP_005386412.1	unique	1519	3	NZ_GG667604.1	1103541	1104305
97	WP_005386421.1	unique	2408	3	NZ_GG667604.1	1104932	1105321
98	WP_005386536.1	unique	2831	3	NZ_GG667604.1	1168880	1169083
99	WP_005387372.1	unique	2581	3	NZ_GG667605.1	147829	148146
100	WP_005387487.1	unique	2182	3	NZ_GG667605.1	204821	205285
101	WP_005387951.1	unique	2727	3	NZ_GG667605.1	496336	496587
102	WP_005387954.1	unique	2268	3	NZ_GG667605.1	496601	497035
103	BBU33667.1	unique	2874	4	AP022321.1	154942	155127
104	BBU33684.1	unique	2735	4	AP022321.1	175139	175387
105	BBU33687.1	unique	2926	4	AP022321.1	176471	176596
106	BBU33733.1	unique	2816	4	AP022321.1	228830	229039
107	BBU33734.1	unique	430	4	AP022321.1	229036	230496
108	BBU33736.1	unique	2626	4	AP022321.1	231027	231323
109	BBU33738.1	unique	836	4	AP022321.1	231602	232732
110	BBU33842.1	unique	558	4	AP022321.1	345807	347138
111	BBU33877.1	unique	2928	4	AP022321.1	379226	379345
112	BBU33988.1	unique	1289	4	AP022321.1	488202	489074
113	BBU33990.1	unique	2907	4	AP022321.1	489674	489835
114	BBU34219.1	unique	40	4	AP022321.1	762673	766407
115	BBU34402.1	unique	2914	4	AP022321.1	968270	968419
116	BBU34408.1	unique	2900	4	AP022321.1	972027	972197
117	BBU34410.1	unique	2310	4	AP022321.1	972713	973135
118	BBU35076.1	unique	2891	4	AP022321.1	1681262	1681441
119	BBU35285.1	unique	2785	4	AP022321.1	1901025	1901249

120	BBU35286.1	unique	2026	4	AP022321.1	1901348	1901884
121	BBU35287.1	unique	2635	4	AP022321.1	1902048	1902341
122	BBU35288.1	unique	2298	4	AP022321.1	1902464	1902889
123	BBU35289.1	unique	2632	4	AP022321.1	1902904	1903200
124	BBU35296.1	unique	2898	4	AP022321.1	1906946	1907116
125	BBU35298.1	unique	2904	4	AP022321.1	1907877	1908041
126	BBU35309.1	unique	1936	4	AP022321.1	1919941	1920513
127	BBU35364.1	unique	63	4	AP022321.1	1972677	1975871
128	BBU35429.1	unique	2755	4	AP022321.1	2045538	2045777
129	WP_004698366.1	unique	2787	5	NC_013520.1	471969	472193
130	WP_012863636.1	unique	348	5	NC_013520.1	30084	31694
131	WP_012863642.1	unique	400	5	NC_013520.1	36098	37606
132	WP_012863755.1	unique	2158	5	NC_013520.1	224086	224559
133	WP_012863780.1	unique	153	5	NC_013520.1	253192	255378
134	WP_012863781.1	unique	821	5	NC_013520.1	255371	256513
135	WP_012863782.1	unique	635	5	NC_013520.1	256541	257812
136	WP_012863783.1	unique	780	5	NC_013520.1	257842	259014
137	WP_012863789.1	unique	904	5	NC_013520.1	262494	263576
138	WP_012863840.1	unique	2817	5	NC_013520.1	341725	341934
139	WP_012863944.1	unique	2788	5	NC_013520.1	471570	471794
140	WP_012863992.1	unique	406	5	NC_013520.1	523510	525000
141	WP_012863994.1	unique	719	5	NC_013520.1	526093	527304
142	WP_012863995.1	unique	91	5	NC_013520.1	527273	529858
143	WP_012863996.1	unique	224	5	NC_013520.1	529865	531781
144	WP_012863997.1	unique	2630	5	NC_013520.1	531786	532082
145	WP_012863998.1	unique	2886	5	NC_013520.1	532564	532743
146	WP_012864001.1	unique	71	5	NC_013520.1	535745	538699
147	WP_012865018.1	unique	195	5	NC_013520.1	2083918	2085933
148	WP_012865019.1	unique	169	5	NC_013520.1	2085997	2088141
149	WP_021148019.1	unique	2742	5	NC_013520.1	864863	865108
150	WP_042466885.1	unique	902	5	NC_013520.1	524993	526075
151	WP_052293367.1	unique	212	5	NC_013520.1	533782	535743
152	WP_071840061.1	unique	2879	5	NC_013520.1	829204	829386
153	WP_080513040.1	unique	2896	5	NC_013520.1	865211	865384
154	PQL15935.1	unique	2833	6	PPCX01000001.1	1	206
155	PQL15936.1	unique	2708	6	PPCX01000001.1	434	694
156	PQL15937.1	unique	2269	6	PPCX01000001.1	816	1250
157	PQL15939.1	unique	2598	6	PPCX01000001.1	1925	2236
158	PQL15941.1	unique	2260	6	PPCX01000001.1	2812	3249
159	PQL15943.1	unique	2143	6	PPCX01000001.1	3482	3958

160	PQL15944.1	unique	2580	6	PPCX01000001.1	4029	4346
161	PQL15949.1	unique	2768	6	PPCX01000001.1	8151	8384
162	PQL15951.1	unique	1924	6	PPCX01000001.1	9918	10493
163	PQL15956.1	unique	2359	6	PPCX01000001.1	14811	15215
164	PQL15957.1	unique	2327	6	PPCX01000001.1	15739	16155
165	PQL15958.1	unique	2849	6	PPCX01000001.1	16986	17180
166	PQL15959.1	unique	2719	6	PPCX01000001.1	17195	17452
167	PQL15960.1	unique	2230	6	PPCX01000001.1	17500	17949
168	PQL15967.1	unique	1684	6	PPCX01000001.1	21882	22565
169	PQL15035.1	unique	1894	6	PPCX01000002.1	35067	35654
170	PQL15037.1	unique	2858	6	PPCX01000002.1	36301	36492
171	PQL14590.1	unique	910	6	PPCX01000003.1	26261	27337
172	PQL14591.1	unique	2205	6	PPCX01000003.1	27406	27861
173	PQL12797.1	unique	1805	6	PPCX01000008.1	37935	38558
174	PQL12798.1	unique	235	6	PPCX01000008.1	38568	40460
175	PQL12800.1	unique	790	6	PPCX01000008.1	42280	43443
176	PQL12801.1	unique	1672	6	PPCX01000008.1	43486	44172
177	PQL12210.1	unique	126	6	PPCX01000010.1	410908	413220
178	PQL12277.1	unique	1026	6	PPCX01000010.1	493791	494795
179	PQL11621.1	unique	2167	6	PPCX01000011.1	82715	83185
180	PQL58287.1	unique	2076	7	PPDD01000005.1	18196	18702
181	PQL57722.1	unique	1204	7	PPDD01000009.1	30374	31297
182	PQL57723.1	unique	65	7	PPDD01000009.1	31303	34467
183	PQL57724.1	unique	50	7	PPDD01000009.1	34471	37929
184	PQL57725.1	unique	1114	7	PPDD01000009.1	37934	38893
185	PQL57726.1	unique	2068	7	PPDD01000009.1	38950	39459
186	PQL57727.1	unique	1621	7	PPDD01000009.1	39468	40184
187	PQL57728.1	unique	2270	7	PPDD01000009.1	40205	40639
188	PQL57729.1	unique	173	7	PPDD01000009.1	40741	42840
189	PQL57602.1	unique	447	7	PPDD01000010.1	136913	138352
190	PQL57259.1	unique	1293	7	PPDD01000012.1	9690	10559
191	PQL57088.1	unique	1999	7	PPDD01000015.1	32864	33409
192	PQL57106.1	unique	234	7	PPDD01000015.1	57671	59563
193	WP_059361140.1	unique	2746	8	NZ_BBXI01000002.1	75110	75352
194	WP_059361161.1	unique	41	8	NZ_BBXI01000002.1	84264	87965
195	WP_059361164.1	unique	1542	8	NZ_BBXI01000002.1	88000	88755
196	WP_059361240.1	unique	2868	8	NZ_BBXI01000002.1	128765	128953
197	WP_059361413.1	unique	2834	8	NZ_BBXI01000002.1	218248	218448
198	WP_059361510.1	unique	2209	8	NZ_BBXI01000003.1	41368	41823
199	WP_059361626.1	unique	1362	8	NZ_BBXI01000003.1	169491	170324

200	WP_036754116.1	unique	2810	8	NZ_BBXI01000009.1	187244	187456
201	WP_059361902.1	unique	2836	8	NZ_BBXI01000009.1	109413	109613
202	WP_059361910.1	unique	2792	8	NZ_BBXI01000009.1	113435	113656
203	WP_059361962.1	unique	1739	8	NZ_BBXI01000009.1	174607	175260
204	WP_059361963.1	unique	187	8	NZ_BBXI01000009.1	175260	177302
205	WP_059361970.1	unique	267	8	NZ_BBXI01000009.1	182852	184645
206	WP_059361978.1	unique	983	8	NZ_BBXI01000009.1	187477	188505
207	WP_059361979.1	unique	313	8	NZ_BBXI01000009.1	188505	190187
208	WP_059361993.1	unique	2132	8	NZ_BBXI01000009.1	196257	196739
209	WP_059362058.1	unique	1937	8	NZ_BBXI01000009.1	231538	232110
210	WP_059362060.1	unique	329	8	NZ_BBXI01000009.1	232111	233757
211	WP_059362067.1	unique	104	8	NZ_BBXI01000009.1	238898	241354
212	WP_059362069.1	unique	835	8	NZ_BBXI01000009.1	241363	242493
213	WP_059364321.1	unique	1869	8	NZ_BBXI01000021.1	138095	138691
214	WP_059364548.1	unique	1973	8	NZ_BBXI01000022.1	19947	20507
215	WP_059364576.1	unique	1011	8	NZ_BBXI01000022.1	39578	40591
216	WP_059364587.1	unique	1241	8	NZ_BBXI01000026.1	1404	2303
