# Supplementary figures and images for: Comparative Pan-Genome Analysis of Oral Veillonella Species
Source: Microorganisms. 2021 Aug 20;9(8):1775. doi: 10.3390/microorganisms9081775 (PMC8400620; doi:10.3390/microorganisms9081775)

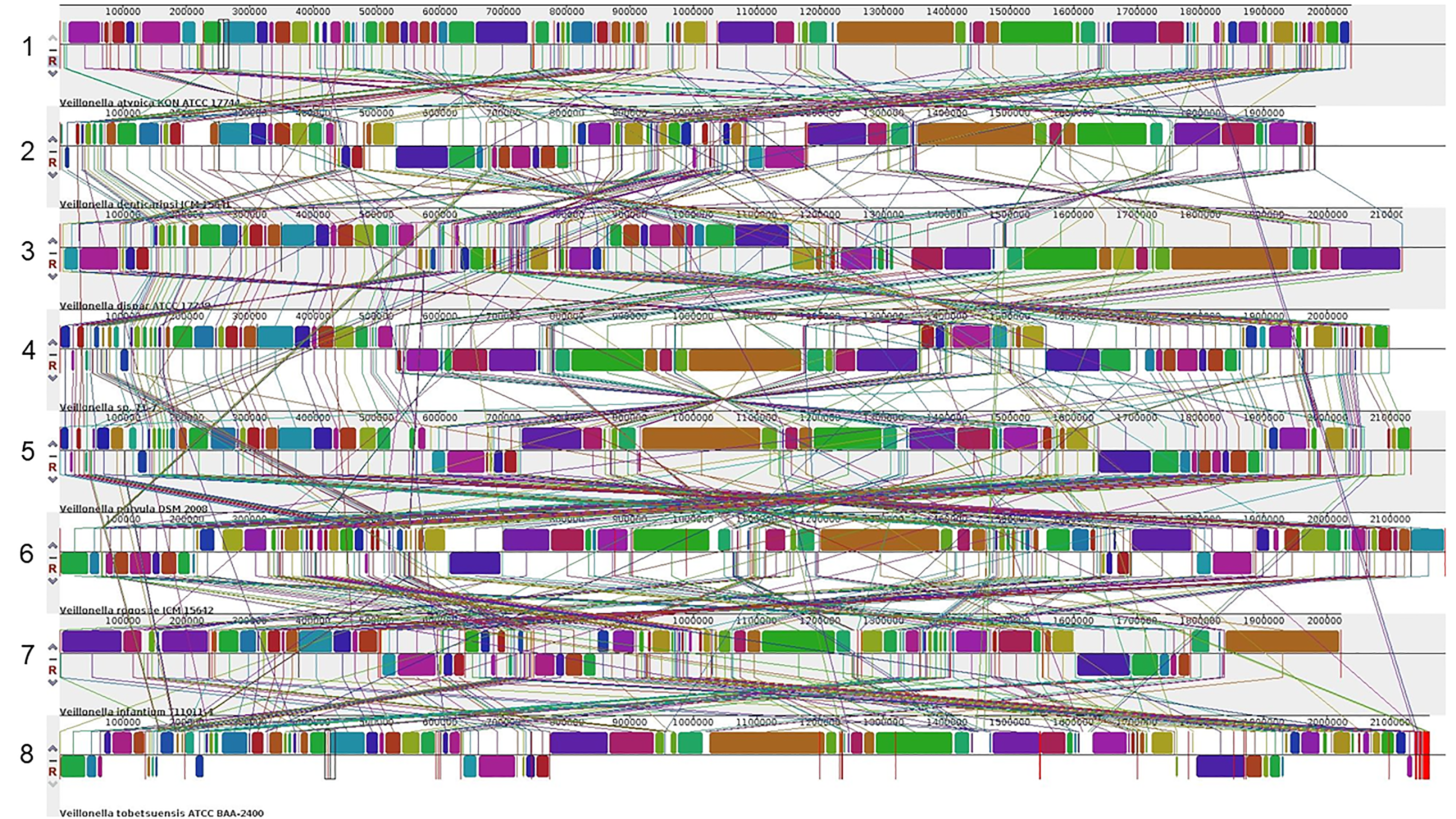

Supplement: Supplementary file 1 [file microorganisms-09-01775-s001.zip › Suppl Figure 1.tif]
